# Recent Advances in Electrochemical Biosensors Based on Fullerene-C_60_ Nano-Structured Platforms

**DOI:** 10.3390/bios5040712

**Published:** 2015-11-23

**Authors:** Sanaz Pilehvar, Karolien De Wael

**Affiliations:** AXES Research Group, Department of Chemistry, University of Antwerp, Groenenborgerlaan 171, 2020 Antwerp, Belgium; E-Mail: sanaz.pilehvar@uantwerpen.be

**Keywords:** nanotechnology, nanostructures, nano-bio hybrids, fullerene-biomolecule, biosensors

## Abstract

Nanotechnology is becoming increasingly important in the field of (bio)sensors. The performance and sensitivity of biosensors is greatly improved with the integration of nanomaterials into their construction. Since its first discovery, fullerene-C_60_ has been the object of extensive research. Its unique and favorable characteristics of easy chemical modification, conductivity, and electrochemical properties has led to its tremendous use in (bio)sensor applications. This paper provides a concise review of advances in fullerene-C_60_ research and its use as a nanomaterial for the development of biosensors. We examine the research work reported in the literature on the synthesis, functionalization, approaches to nanostructuring electrodes with fullerene, and outline some of the exciting applications in the field of (bio)sensing.

## 1. Introduction

Bio-nanotechnology is a new emerging field of nanotechnology and combines knowledge from engineering, physics, and molecular engineering with biology, chemistry, and biotechnology aimed at the development of novel devices, such as biosensors, nanomedicines, and bio-photonics [1]. A biosensor is an analytical device that consists of a biological recognition element in direct spatial contact with a transduction element which ensures the rapid and accurate conversion of the biological events to measurable signals [2]. However, the discovery of rich nanomaterials has opened up new opportunities in the field of biosensing research and offer significant advantages over conventional biodiagnostic systems in terms of sensitivity and selectivity [2,3].

Among various nanostructure materials, carbon nanomaterials have been receiving great attention owing to their exceptional electrical, thermal, chemical, and mechanical properties and have found application in different areas as composite materials, energy storage and conversion, sensors, drug delivery, field emission devices, and nanoscale electronic components [4,5,6]. Moreover, the possibility to customize their synthesis with attached functional groups or to assemble them into three-dimensional arrays has allowed researchers to design high surface area catalysts and materials with high photochemical and electrochemical activity. Their exceptional electrochemical properties lead to their wide application for designing catalysts for hydrogenation, biosensors, and fuel cells [6]. The wide application of carbon nanomaterial for construction of biosensors is partly motivated by their ability to improve electron-transfer kinetics, high surface-to-volume ratios, and biocompatibility [6,7]. In addition, the use of nanomaterials can help to address some of the key challenges in the development of biosensors, such as sensitive interaction of an analyte with biosensor surface, efficient transduction of the biorecognition event, and reduced response times.

Various kinds of zero-, one-, two-, and three dimensional carbon nanomaterials have been used. Examples of such materials include carbon nanotubes, nanowires, nanoparticles, nanoclusters, graphene, *etc.* [8]. Fullerenes are a very promising member of carbon nanostructure family. The closed cage, nearly-spherical C_60_ and related analogues have attracted great interest in recent years. Multiple redox states, stability in many redox forms, easy functionalization, signal mediation, and light-induced switching are among their exceptional properties. In different applications, fullerenes have been used for the development of superconductors, sensors and biosensors, catalysts, optical and electronic devices [9,10]. Their superior electrochemical characteristics combined with unique physiochemical properties enable the wide application of fullerenes in the design of novel biosensor systems [11]. It is the aim of this review to present the most recent and relevant contributions in the development of biosensors based on fullerene-C_60_ and different biological components. A brief introduction and history of fullerene-C_60_ is first presented in Section 1.2. Available methods for synthesis and functionalization of fullerene-C_60_ are mentioned in Section 1.3. Finally, we briefly outline the current status and future direction for electrochemical biosensors based on fullerene-C_60_, especially, fullerene-C_60_ as a immobilizing platform for DNA. Recently, Afreen *et al.* introduced a review on functionalized fullerene-C_60_ as nanomediators for construction of glucose and urea biosensors [12]. However, the present review covers all aspects of biosensors based on fullerene-C_60_.

### 1.1. Basics and History of Fullerene (C_60_)

**Figure 1:**
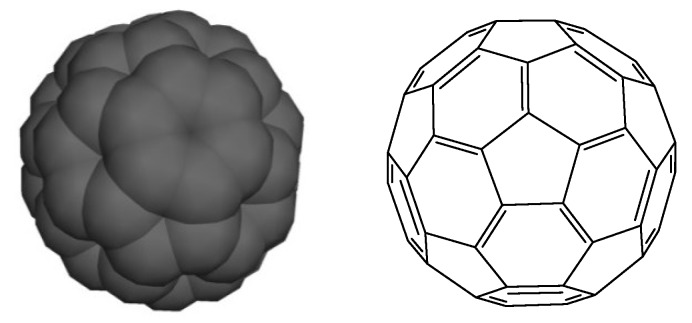
Schematic representation of C_60_ [1].

Fullerene is built up of fused pentagons and hexagons forming a curved structure. The smallest stable, and the most abundant, fullerene obtained by the usual preparation method is the I_h_-symmetrical buckminsterfullerene C_60_. The next stable homologue is C_70_ followed by higher fullerenes C_74_, C_76_, C_78_, C_80_, C_82_, C_84_, and so on [13,14]. Since the discovery of fullerenes, buckminsterfullerene (C_60_) has fascinated a large number of researchers due to its remarkable stability and electrochemical properties. The stability of the C_60_ molecules is due to the geodesic and electronic bonding present in its structure (Figure 1). In 1966, Deadalus (also known as D.E.H Jones) considered the possibility of making a large hollow carbon cage (giant fullerene). Later on, in 1970, Osawa first proposed the spherical I_h_-symmetric football structure for the C_60_ molecule. In 1984, it was observed that upon laser vaporization of graphite large carbon clusters of C_n_ with *n* = 30–190 can be produced. The breakthrough in the discovery of the fullerene happened in 1985 when Kroto and Smalley proved the presence of C_60_ and C_70_ which can be produced under specific clustering conditions. The second breakthrough in fullerene research was achieved by Kratschmer and Huffman. They invented the laboratory analogues of interstellar dust by vaporization of graphite rods in a helium atmosphere and observed that upon choosing the right helium pressure, the IR spectrum shows four sharp strong absorption lines which were attributed to C_60_ [11,15]. 

Each carbon in a fullerene-C_60_ atom is bonded to three others and is sp^2^ hybridized. The C_60_ molecule has two bond lengths, the 6:6 ring bonds can be considered as double bonds and are shorter than the 6:5 bonds. C_60_ is not “superaromatic” as it tends to avoid double bonds in the pentagonal rings, resulting in poor electron delocalization. Therefore, C_60_ structure behaves like an electron-deficient alkene, and reacts readily with electron-rich species. The estimated values of electron affinity (EA) (2.7 eV) and ionization potential (IP) (7.8 eV) of C_60_ indicate that it can easily contribute to the electron transfer reaction and reveal very rich electrochemistry which makes them attractive candidates for electroanalytical applications [7,11,16].

### 1.2. Synthesis of Fullerene

It was initially shown that the production of fullerene is achievable by means of an irradiating laser beam on a graphite rod placed in a helium atmosphere [17]. However, the overall yield rate of fullerene was insufficient for its potential applications in various industrial fields. Therefore, different production methods have been developed for sufficient production of fullerene [18,19,20]. The second proposed method is based on laser ablation of graphite in a helium atmosphere. In the laser ablation method, materials are removed from a solid surface by irradiating it with a laser beam. During the laser irradiation of graphite, materials are evaporated and their vapors are converted to plasma. Upon cooling the gas, the vaporized atom tends to combine and form fullerene [17]. The arc discharge process is an alternative method, where the vaporization of the input carbon source is achieved by the electric arc formed between two electrodes [17,18]. They can also be produced by the non-equilibrium plasma method, where a non-equilibrium gas phase in the glow discharge is induced by the non-equilibrium plasma and fullerene is generated without the need for high temperatures [21]. 

### 1.3. Functionalization of Fullerene

**Scheme 1:**
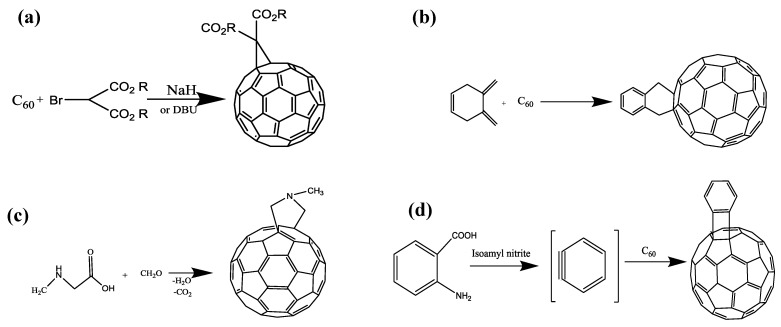
(**a**) The Bingel reaction; (**b**) the Diels-Alder reaction; (**c**) the Prato reaction; and (**d**) the Cycloaddition reaction.

Early studies on the C_60_ molecular structure showed that this carbon allotrope could undergo electron deficient polyolefin reactions. The [6,6] bonds have greater double bond character and are shorter than the [5,6] bonds and, thus, used to functionalize C_60_ by nucleophilic, radical additions, as well as cycloadditions [22]. Many reactions have been developed for the functionalization of C_60_, which consists of cyclopropanation (the Bingel reaction), [4+2] cycloaddition (the Diels-Alder reaction) and [3+2] cycloaddition (the Patro reaction), [2+2] cycloaddition. The Bingel reaction has been frequently used to prepare C_60_ derivatives in which a halo ester or ketone is first deprotonated by a base and subsequently added to one of the double bonds in C_60_ resulting in an anionic intermediate that reacts further into a cyclopropanated C_60_ derivative. In addition, cyclopropanation reactions have shown to be an efficient method for the preparation of fullerene derivatives with wide application in material science and biological applications (Scheme 1a) [23,24]. Additionally, the double bonds exist in C_60_ can react with different dienes by Diels-Alder reaction (Scheme 1b). The main drawback of Diels-Alder reaction is low thermal stability of formed product [23,25]. In another reaction (the Prato reaction), an azomethine ylide, generated *in situ* by the decarboxylation of iminium salts derived from the condensation of α-amino acids with aldehydes or ketones, react with fullerene to produce [3+2] cycloadduct (Scheme 1c) [23]. A wide variety of functionalization of fullerene molecule is possible by means of the Patro reaction. In another way, addition of benzyne to C_60_ leads to the formation of [2+2] cycloadducts (Cycloaddition) (Scheme 1d) [26]. However, among available methods for functionalization of fullerene, cycloaddition reactions have emerged as very useful for functionalization of one or several of the fullerene double bonds.

## 2. Modification of Electrodes with Fullerenes

The idea of introducing C_60_ chemically-modified electrodes (CME) was first reported by Compton and co-workers in 1992 [27]. They prepared C_60_-based CMEs by immobilizing C_60_ films by drop coating onto surfaces of the electrodes, which were then coated with Nafion as protecting films. It was observed that the current signal improved compared to those using C_60_ dissolved in solution. Afterwards, the electrochemical behavior of the C_60_-CMEs, in non-aqueous and aqueous solutions, has been widely investigated, suggesting the possibility of their electroanalytical applications [28]. Currently, fullerene-based CMEs are prepared in several different ways. The most common method is drop coating the electrode by using a fullerene solution of a volatile solvent [29,30,31]. Electrochemical deposition is another technique of the fullerene film preparation [32]. Moreover, fullerene-based CMEs can be prepared by electro-polymerization where the formed fullerene units are connected by polymer side chains or via epoxide formation [33]. Alternative method for C_60_ films preparation is the self-assembled monolayer (SAM) films using either thiols or silane derivatives of C_60_ on the electrode surfaces [34]. 

In addition, fullerene-C_60_ are widely used for construction of electrochemical biosensors. Generally, electrochemical biosensors are analytical devices which consists of a bioreceptor, an electrochemical active interface, a transducer element which convert biological reaction to a electrical signal, and a signal processor [35]. The principle of the electrochemical biosensors is based on the specific interaction between the analyte and biorecognition element which is also associated with better correlation between the bioreceptor and the transducer surface [36,37,38]. Utilization of different kinds of nanomaterials leads to the important improvements in these aspects and nanomaterials integrate widely in the construction of biosensors in order to improve the sensing performance of the biosensors [2]. The ability of signal mediation, easy functionalization, and light-induced switching lead to the fact that fullerene be considered as new and attractive element in the fabrication of biosensors. 

**Figure 2:**
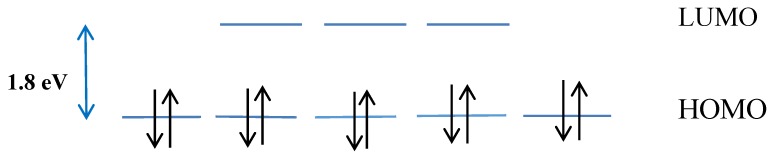
HOMO and LUMO gap in fullerene-C_60_.

Different biomolecules or organic ligands can be immobilized to the shell of fullerenes by adsorption or covalent attachment [39]. Taking into account that fullerenes are not harmful to biological material and they are small enough, they can locate the closest distance to the active site of biomolecules and easily accept or donate electrons to the species surrounding it and make close arrangements with biomolecules [9,14]. Furhermore, they are an ideal substrate for absorbing energy, taking up electrons and releasing them with ease to a transducer. Their high electron-accepting property is due to a low-lying, triply-degenerate, lowest unoccupied molecular orbital (LUMO) which is around 1.8 eV above its five-fold degenerate highest occupied molecular orbital (HOMO) (Figure 2) [40].

Carbon nanotubes (CNT) were also widely used to chemically functionalized electrodes due to their remarkable electrical, chemical, mechanical, and structural properties. It was shown that CNT which is chemically modified with different functionalized groups would make the electrodes more sensitive and selective in detection applications. Furthermore, CNTs have advantages over other carbon nanomaterials, as they exhibit superior electrocatalytic properties [28].

### 2.1. Fullerene(C_60_)-DNA Hybrid

In general, combining two or more different materials via interaction forces leads to the appearance of novel hybrid materials with unique properties. Taking advantage of nanoscale properties of biomolecules hold great promise, since their characteristic is different from their bulk complements. Biomolecules hold great compatibility and suitability to form bio-nano hybrid structures and significant efforts have been made to form the nano-bio hybrid systems for various applications [41,42]. There are various approaches available to create hybrid materials consist of biomacromolecules and nanomaterials. One approach is based on connecting the molecules through covalent bonding and the other approach is adsorption (or wrapping) of a material onto the surface of the other materials via supramolecular interactions, encapsulation or groove of the other molecule [43,44,45].

Among the various biomolecules, DNA attracted a great attention due to its superior properties such as structural regularity, biocompatibility, and unique double helix structure, which leads a range of outstanding properties that are hard to find in other biomolecules [46]. Furthermore, DNA is a potential material for combining with other chemicals, especially with nanomaterials by different interactions. Taking into account the advantages of DNA and carbon nanomaterials, the combination of DNA and carbon nanomaterials offers unique advantages for different application. Hence, the combination of DNA with new carbon allotropes is a skillful and challenging area that can lead to the development of novel nano-biomaterials with exceptional properties for a variety of potential applications such as gas sensors and catalysts, as well as electronic and optical devices, sensitive biosensors, and biochips [46,47].

#### 2.1.1. Interaction of DNA with Fullerene

The interaction between DNA and other species plays an important role in life science since it is in direct contact with the transcription of DNA, mutation of genes, origins of diseases, and molecular recognition studies [45]. Cassell *et al.* studied the interaction between DNA and fullerene. C_60_-*N*,*N*-Dimethylpyrrolidinium iodide is used as a complexing agent to form DNA/fullerene complexes through phosphate groups of the DNA backbone, which was imaged by TEM. It was shown that the complexation of free fullerene with DNA is sterically permitted and surfactants can be used in order to prevent the DNA/fullerene hybrids from aggregation [48]. 

Later on, Pang *et al.* [49] studied the interaction of DNA with fullerene-C_60_ in depth. The method used was based on the double-stranded DNA (dsDNA) modified gold electrodes (dsDNA/Au) in combination with electrochemical method for investigation of the interactions between C_60_ derivatives and DNA. They have chosen [Co(phen)_3_]^3+/2+^ as an appropriate electroactive indicator, which can interact electrostatically and intercalatively with dsDNA, to characterize the interactions. In the presence of dsDNA, the peak currents for [Co(phen)_3_]^3+/2+^ decreased due to its interaction with dsDNA and then recovered significantly in the presence of H_10_C_60_(NHCH_2_CH_2_OH)_10_. Electrochemical studies with dsDNA-modified gold electrodes suggested that the C_60_ derivative could interact strongly with dsDNA, with binding sites of the major groove of the double helix and phosphate backbone of dsDNA. The interaction between dsDNA and H_10_C_60_(NHCH_2_CH_2_OH)_10_ was attributed to the interaction between the delocalized π electrons of H_10_C_60_(NHCH_2_CH_2_OH)_10_ and DNA and the binding of H_10_C_60_(NHCH_2_CH_2_OH)_10_ to the major groove of the double helix as well. It is believed that H_10_C_60_(NHCH_2_CH_2_OH)_10_, in the protonated form, interacts electrostatically with the negatively-charged phosphate backbone of the dsDNA. It can also access the major groove of the double helix and interact with the delocalized π system of bases of dsDNA. When fullerene is electrically neutral, the electrostatic interaction with the dsDNA vanishes and the π- π interactions is present. In addition, it was shown that the binding and dissociation of H_10_C_60_(NHCH_2_CH_2_OH)_10_ to the dsDNA is a reversible process [49].

So far, it was believed that the water-soluble C_60_ molecules only bind to the major grooves and free ends of the double-strand DNA, but extensive simulations indicated that the association of hydrophobic C_60_ can also occur at the minor groove sites and no complexation occurs at the major grooves. It is stated that the free ends of the double-strand DNA fragment are the hydrophobic regions which favor the diffusion of hydrophobic fullerenes toward their docking sites [50]. Calculation of binding energy showed that the hybrid C_60_-DNA complexes are energetically favorable compared to the unpaired molecules. The self-association of C_60_ molecules in the presence of DNA molecules revealed that self-association between C_60_ molecules occurs in the early stages of simulation. However, after 5 ns of simulation, one of the C_60_ molecules binds to one end of the DNA. Visual observation of the obtained results from simulation showed that the overall shape of the dsDNA molecule is not affected by the association of C_60_. However, the association C_60_ has more impact on the DNA structure when more hydrophobic contacting surfaces are exposed at the end of double-strand DNA. The binding between the C_60_ and DNA molecules is attributed to the hydrophobic interaction between the C_60_ and hydrophobic sites on the DNA [50]. In another study, it is reported that the C_60_ molecule binds to the ssDNA molecule with a binding energy of about −1.6 eV, and the results are in close agreement with those given in the previous references [51]. However, it is stated that the mobility of DNA and their interaction with water molecules which often present in real physical systems were not taking into these calculations.

#### 2.1.2. Fullerene for DNA Biosensing

The preparation of DNA hybridization sensors involves the attachment of oligonucleotide probes on the surface of electrode, and DNA immobilization step has been considered as a fundamental step in fabrication of DNA biosensing [52,53]. Various electrode materials, such as gold, carbon paste, glassy carbon, carbon fibers, and screen printed electrodes, have been utilized to immobilize the DNA. Despite, carbon nanomaterials such as C_60_ are compounds that have attracted much interest as the materials for DNA sensors and biosensors because of their unique properties. Shiraishi *et al.* demonstrated a new procedure of immobilizing DNA onto a fullerene impregnated screen printed electrode (FISPE) for detection of 16S rDNA, extracted from *Escherichia coli* [54]. The integrated FISPE was the mixture of ink and fullerene solution which is modified with probe DNA in the next step. The efficiency of the developed method was tested by detecting 46S rDNA of *E. coli* by means of the modified electrode with perfectly matched probes. It is shown that the reduction peak of Co(phen)_3_^3+^ is enhanced only on the perfectly matched probes modified electrode after hybridization. This fact was ascribed to the accumulation of indicator into the hybrid between perfectly matched probe and rDNA of target. In addition, it was observed that the electrochemical response of Co(phen)_3_^3+^ accumulated in the hybrid was better when using FISPE which based on the authors opinion shows that the probe DNA was immobilized onto the PA-FISPE surface in a high concentration.

Other carbon nanomaterials were also used for the development of new (bio)sensing systems for applications in the food industry, environmental monitoring, and clinic diagnostics. For example, recently CNT-modified arrays have been used to detect DNA targets by combining the CNT nanoelectrode array with Ru(bpy)^2+^_3_ mediated guanine oxidation [55]. In another study, a MWCNT-COOH-modified glassy carbon was used in combination with an amino functionalized oligonucleotide probe and pulse-voltammetric transduction [56]. Recently, an indicator-free AC impedance measurements of DNA hybridization based on DNA probe-doped polypyrrole film over a MWCNT layer reported by Cai *et al.* [57] A five-fold sensitivity enhancement was observed compared to analogous measurements without CNT. However, most of the examples suffer from the feasibility of scale up conditions due to the low yield and expensive experimental procedures. Inhomogeneity in CNT samples due to the different production procedures, limits their application.

#### 2.1.3. Fullerene as an Immobilization Platform

Nanosized materials can be used as potential building blocks to construct higher ordered supramolecular architectures for designing the highly-sensitive biosensing platform. The working electrode modified with partially reduced fullerene-C_60_ modified electrode had exceptional properties, such as high electroactive surface area, excellent electronic conductivity, and good biocompatibility [58,59]. Zhang *et al.* have developed a technique to disperse fullerene C_60_ nanotubes (FNTs) homogenously into aqueous solution by forming a kind of complex with ssDNA [58]. The FNT/DNA was modified onto the surface of the GCE by air-drying/adsorption, enabling the electrochemical analysis of the modified electrode with voltammetric technology. The electrochemical detection of dopamine (DA) in the presence of ascorbic acid was performed. The interaction of FNT with DNA was studied by UV–Vis measurements. The observed red shift attributed to the weak binding between the two, and it was shown that π–π stacking and hydrophobic interaction contribute in the formation of FNT/DNA hybrid. It is believed that the strong physisorption of DNA onto the FNTs via a wrapping mechanism prevent the FNT/DNA from precipitation upon adding water or organic solvent. Obtained SEM images of the surface FNT/DNA modified electrode proved the formation of uniform films. 

In another study, Gugoasa *et al.* investigated the influence of dsDNA which is physically immobilized on the multi-walled carbon nanotubes (MWCNT), synthetic monocrystalline diamond (DP) and fullerenes-C_60_ on the detection of three different neutransmitters such DA, epinenephrine and norepinephrine [60]. Optimized working condition for dsDNA biosensors was found to be a value of 4 for pH and the 0.1 mol/L KNO_3._ It has been shown that the highest improvement of the signal for the DA was recorded when dsDNA was immobilized on DP. However, the larger working concentration and the lowest limit of detection were obtained when dsDNA has been immobilized on MWCNT. In addition, immobilization of dsDNA on fullerene-C_60_ decreases both the limit of detection and the limit of quantification. This occurrence attributed to the fact that the immobilization matrix has a very important contribution to the biosensor performance. It was shown that not only the nature of the material, but also the geometry of the substances at the molecular level has the effect on the behavior of the biosensors [60].

**Scheme 2:**
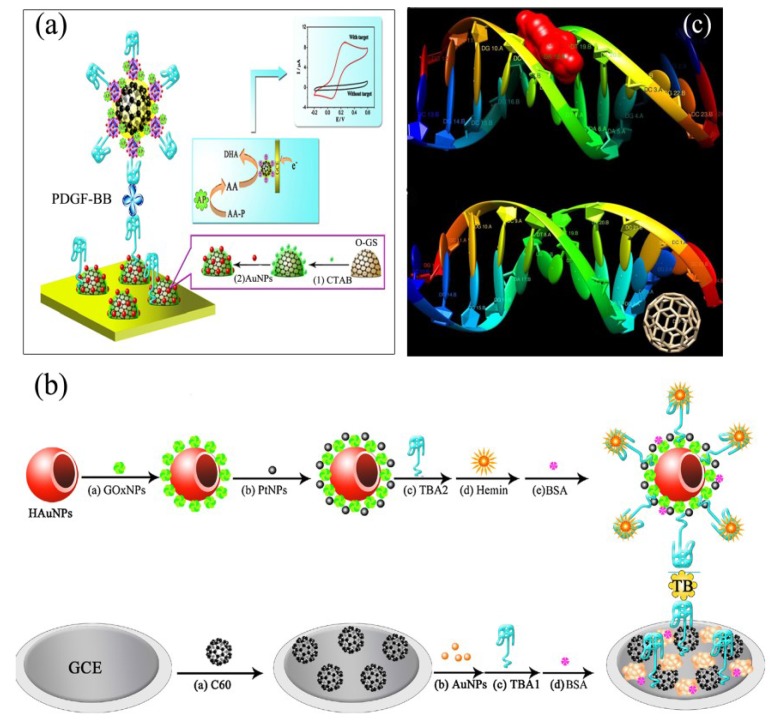
(**a**) Schematic illustration of the stepwise aptasensor fabrication process and the dual signal amplification mechanism, adapted from [64]; (**b**) schematic diagram of fabrication and detection of the ECL aptasensor, adapted from [65]; and (**c**) results of molecular modeling related to (**A**) groove binding of small molecules to the minor groove of dsDNA and (**B**) groove binding of fullerene-C_60_ to the major groove of dsDNA, adapted from [66].

However, the obtained fullerene-C_60_ by simply stirring or ultrasonication treatments was not suitable for biomedical applications because of their aggregation properties. To solve these limitations, the covalent binding of nano-C_60_ to aminoacids, hydroxyl groups, carboxyl groups *etc.*, which can increase the nanoparticle’s ability to interact with the biological environment can be performed [61,62,63]. On the other hand, synthesis of the functionalized C_60_ with non-covalent interaction based on supramolecular chemistry would preserve the original structure and electrochemical properties of C_60_. Supramolecular chemistry is the chemistry of the intermolecular bond, aims at developing highly complex chemical system components in interacting by non-covalent intermolecular forces [64]. A new supramolecular method is developed by Han *et al.* for preparation of thiol and amino functionalized C_60_ nanoparticles with better water solubility and larger active surface area [64]. They used amino functionalized 3,4,9,10-perylenetetracarboxylic dianhydride (PTC-NH_2_) as a π electron compound which can be bond to the surface of C_60_ via supramolecular interaction. Prussian blue carried gold nanoparticles (Au@PBNPs) were interacted with FC_60_NPs. In the next step, the detection aptamers for platelet-derived growth factor B-chain (PDGF-BB) as a model target was labeled by Au@PB/FC_60_ and the coupled with alkaline phosphatase (AP) for electrochemical aptasensing (Scheme 2a). The combination of fullerene-C_60_ and AuNPs have been used for immobilization of a large amount of capture aptamers on the surface of electrode. The obtained SEM and TEM images showed that the Au@PBNPs were adsorbed uniformly and tightly on the FC_60_NPs. The performance of developed aptasensor was investigated by detecting PBGF-BB standard solutions (Table 1).

Electrochemiluminescence (ECL) is a powerful analytical tool for the detection of clinical samples. A peroxydisulfate/oxygen (S_2_O_8_^2−^/O_2_) system is widely used for amplification of ECL signals where the dissolved O_2_ can serve as a co-reactant [67]. The enzymatic reaction can catalyze *in situ* generation of the dissolved O_2_ [63,65]. Zhao *et al.* [65] developed a sandwich-type aptasensor based on mimicking bi-enzyme cascade catalysis to *in situ* generate the co-reactant of dissolved O_2_ for signal amplification to detect thrombin (TB). Au nanoparticles (HAuNPs) were utilized as carriers to immobilize glucose oxidase nanoparticles (GOxNPs) and Pt nanoparticles (PtNPs). GOxNPs could catalyze the glucose to generate H_2_O_2,_ which could be further catalyzed by hemin/G-quadruplex and PtNPs, in order to *in situ* generate dissolved O_2_ with high concentration. In this study, the detection aptamer of thrombin (TBA2) was immobilized on the PtNPs/GOxNPs/HAuNPs and hemin was intercalated into the TBA2 to obtain the hemin/G-quadruplex/PtNPs/GOxNPs/HAuNPs nanocomplexes, which was utilized as signal tags (Scheme 2b). The surface of glassy carbon is modified with C_60_ and electrochemical deposited Au nanoparticles for further immobilization of thiol-terminated thrombin capture aptamer (TBA1). The TBA1, TB, and TBA2 make a sandwich-type structure. The zero-dimensional nano-C_60_ was shown to enhance the immobilization of nanoparticles but also amplified the ECL signal owing to its large specific surface area. The developed aptasensor is characterized by the ECL measurements. The bare GCE showed relatively low ECL intensity in the low concentration level of dissolved O_2_. The ECL intensity of the bare GCE was enhanced in the presence of dissolved O_2_. The ECL intensity was increased when using nano-C_60_ was coated onto the electrode, due to the enrichment effect of nano-C_60_ on peroxydisulfate luminescence. Electrodepositing of AuNPs was further enhance the ECL intensity since it accelerate the electron transfer in ECL reaction. However, the ECL intensity decreased successively when TBA1 were immobilized onto the electrode. The ECL signal dropped again after the incubation of modified electrode with the target analyte of TB. The ECL aptasensor also evaluated by CV in 0.1 M PBS. While the relatively low CV intensity was obtained at bare GCE, the CV intensity reduced when electrode was coated with C_60_ due to its low electrical conductivity (Table 1).

In another report, Gholivand *et al.* studied the mechanism of the prevention of Parkinson’s disease by means of Carbiodopa (CD) drug at a double-stranded DNA (dsDNA) and fullerene-C_60_-modified glassy carbon electrode (dsDNA/FLR/GCE) by cyclic voltammetry [66]. They have used multivariate analysis to distinguish the complex system. Firstly, the effect of pH on the electrochemical system has been studied and a value of 4.0 for pH resulted in higher sensitivity of the system. It has been shown that the oxidation of CD was controlled by adsorption at the dsDNA/FLR/GCE. In addition, the CV recorded at different electrodes showed that the electrocatalytic behavior for oxidation of CD at FLR/GCE is improved noticeably in comparison with the bare GCE. When dsDNA was added to the CD solution both oxidation and reduction peaks decreased markedly and shifted to less and more positive potentials, respectively, which indicate that CD interacts with dsDNA. They have been used electronic UV–Vis absorption spectroscopy to characterize the interaction between dsDNA and small molecules. In the obtained spectra, no redshift was observed, which represents that the binding mode is not the intercalative binding and it could be groove binding [66]. By means of all these observations, it has been suggested that small molecules, such as CD, interact with the minor groove, while large molecules (fullerene-C_60_) tend to interact with the major groove binding site of DNA. This phenomenon was earlier reported and further proved by molecular modeling which is performed in this study (Scheme 2c). 

### 2.2. Fullerene(C_60_)-Antibody Hybrid

Conventional immunosensors suffer from drawbacks, such as intrinsic complexity and the requirement for signal amplification, large sample size, and high cost. By using nano-scale carbon materials, most of these limitation can be solved [68]. Especially, fullerene C_60_ with conjugate π electrons can be considered as electrophilic molecules, which can be attacked by electron-donating molecules, such as amines, antibodies, and enzymes. 

**Scheme 3:**
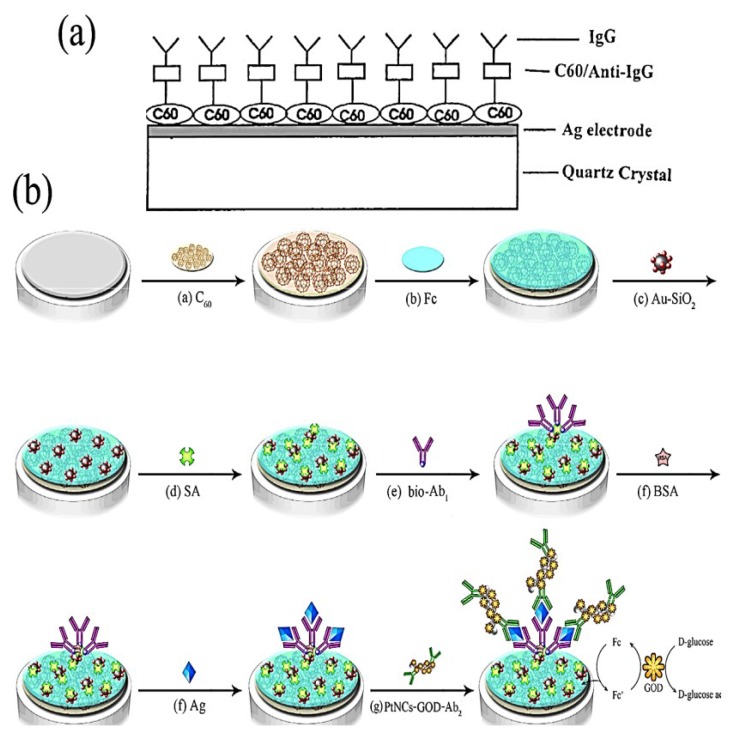
(**a**) Diagrams of the C_60_-anti-human IgG-coated quartz crystal electrode for IgG, adapted from [69]; and (**b**) the fabrication of the electrochemical immunosensor for *Escherichia coli* O157:H7, adapted from [72].

A sensitive immobilized C_60_-antibody-coated piezoelectric crystal sensor, based on C_60_-anti-human IgG and C_60_-anti-hemoglobin, were developed to detect IgG and hemoglobin in aqueous solutions (Scheme 3a) [69]. For this purpose, a fullerene C_60_-coated piezoelectric quartz crystal has been used to investigate the interaction between C_60_ and the antibody and the change in the resonant frequency of the crystal is recorded which is directly related to the deposited mass [70]. The frequency change responds sensitively to the adsorption of anti-IgG onto the C_60_ coated crystals. The interaction between C_60_ and anti-IgG is found to be chemisorption with good reactivity. The effect of the C_60_ coating load on the frequency response of the C_60_ coated PZ crystal for anti-IgG in water was investigated. The PZ quartz crystal with more C_60_ coating exhibited a larger frequency shift, but the frequency shift of the C_60_-coated PZ sensor tends to level off with larger amounts of C_60_ coating suggesting that C_60_ can only adsorp IgG on its surface to some extent. The obtained results has been revealed that the concentration of antibody, temperature, and pH have an impact on the response of the biosensor. 

The immobilized C_60_-anti-hemoglobin (C_60_-Hb)-coated piezoelectric quartz crystal hemoglobin bio-sensor was also developed to detect hemoglobin in solutions. The partially irreversible response of the C_60_-coated piezoelectric crystal for anti-hemoglobin was tested, suggesting the chemisorption and the good reactivity of anti-hemoglobin on C_60_ coated crystal. The immobilized C_60_-Hb coated piezoelectric crystal sensor exhibited linear response frequency to the concentration of hemoglobin with sensitivity of about 1.56 × 10^4^ Hz/(mg/mL) and detection limit of <10^−4^ mg/mL to hemoglobin in solutions (Table 1) [71].

Recently, Li *et al.* reported the development of a sensitive and efficient electrochemical immunosensor for amperometric detection of *Escherichia coli* O157:H7 (*E. coli* O157:H7) [72]. The immunosensing platform was first composed of fullerene, ferrocene, and thiolated chitosan composite nano-layer (C_60_/Fc/CHI–SH) and then Au nanoparticle-coated SiO_2_ nanocomposites were assembled on the thiolated layer. Next, the large amount of avidin was coated on the Au-SiO_2_ surface, which was used to immobilize biotinylated capture antibodies of *E. coli* O157:H7 (bio-Ab1). For signal amplification, the glucose oxidase (GOD)-loaded Pt nanochains (PtNCs) were used as a tracing tag to label signal antibodies (Ab2) (Scheme 3b). It has been shown that Au-SiO_2_ embedded C_60_/Fc/CHI–SH provide a biocompatible platform for increasing the surface area to capture a large amount of SA/bio-Ab1 and Ab2 and GOD multi-functionalized PtNCs nanocomposites as amplified signals.

### 2.3. Fullerene(C_60_)-Protein Hybrid

Direct electron transfer of biological redox proteins plays an important role in elucidating the intrinsic thermodynamic characteristics of biological systems and designing new kinds of biosensors or biomedical devices [73]. Fullerenes (C_60_) are ideal nanomaterial for absorbing energy, taking up electrons and releasing them to the transducer. They are small enough to locate at closest distance to the active site of the catalytic enzyme, which makes the electron transfer easier. Moreover, they are not harmful to biological material and proteins [74,75,76]. The interaction between the enzyme and the nanomaterial surface could be a covalent or non-covalent bond. The improved stability, accessibility, and selectivity, as well as the reduced leaching, can be achieved through covalent bonding because the location of the biomolecule can be controlled [77]. Moreover, several types of immobilization methods have been developed for biomolecules. These methods include entrapment, encapsulation, covalent binding, cross-linking, and adsorption [78,79].

#### 2.3.1. Enzymes

*Glucose oxidase:* The determination of glucose is medically important for diagnosis of diabetes since the low absorption of glucose can lead to diabetes. In general, glucose is being detected by an electrochemical method with an immobilized glucose oxidase (GOD) enzyme. Glucose oxidase (GOD) is a glycoprotein which catalyzes the electron transfer from glucose to oxygen with the byproduct of gluconic acid and hydrogen peroxide [80,81,82]. The preparation of the immobilized GOD enzyme surface is a crucial step in the development of electrochemical glucose sensors. In most cases, GOD enzymes were immobilized by the entrapment of GOD in polymers or macromolecules, e.g., polyvinyl alcohol, agar, collagen, cellulose triacetate, gelatin, and Nafion [83,84,85]. On the other hand, the covalently-coupled enzymes results in the formation of highly stable bonds between enzyme and matrix [86]. Electron-releasing molecules such as amines can attack fullerene-C_60_ with 60 π electrons. Therefore, the NH group containing enzyme molecules is expected to bond chemically to the fullerene C_60_ molecule, resulting in the formation of stable, immobilized C_60_-enzymes [82].

Chuang *et al.* reported fullerene C_60_-glucose oxidase immobilized enzyme platform to catalyze the oxidation of glucose and produce gluconic acid which was detected by a C_60_-coated PZ quartz crystal sensor for glucose [87]. Fullerene C_60_ was used as a coating material on the quartz crystal of a PZ crystal glucose sensor. The C_60_-glucose oxidase platform was characterized by FT-IR spectroscopy with absorption peaks at 1148 cm^−1^ and 1600 cm^−1^ of glucose oxidase and 525–570 cm^−1^ for fullerene C_60_. The activity of the synthetic C_60_-glucose oxidase was investigated by means of the oxygen electrode detector and to catalyze the oxidation of glucose, which results in the consumption of oxygen. The effect of the amount of the immobilized glucose oxidase on the oxidation rate was investigated. It was shown that the consumption of oxygen is linearly proportional to the number of pieces of immobilized enzyme. The obtained results showed that only C_60_ coated crystals with immobilized enzyme responded sensitively to glucose. The studies on the effect of the amount of C_60_ coating on the frequency response of the PZ glucose sensor with the immobilized enzyme C_60_ glucose oxidase shows that the thicker C_60_ coating exhibits a better response but, with a larger amount of coating, it is leveled off. The pH and temperature effect on the activity of the immobilized enzyme C_60_-glucose showed that an optimum pH of 7 and 30 °C for temperature is suitable for the glucose oxidase activity (Table 1). 

In another report, direct electrochemistry of glucose oxidase (GOD) was achieved with GOD-hydroxyl fullerenes (HFs) modified glassy carbon electrode which protected with a chitosan membrane [80]. The formed GOD-HFs nanoparticles in the chitosan membrane was characterized with TEM images, which showed the average size of 20 nm for GOD-HFs nanoparticles. It has been shown that while no redox peak was observed at bare was bare GCE, Chit/GOD/GCE and Chit/HFs/GCE, a pair of well-defined redox peaks was observed at the Chit/GOD-HFs/GCE. The CVs remained unchanged after successive potential cycle, showing that the formed Chit/GOD-HFs was stable on the GC electrode. In addition, the obtained $K_{m}^{app}$ value was lower than that of conventional values which showing a strong interaction and higher affinity of glucose for the modified electrode. 

**Scheme 4:**
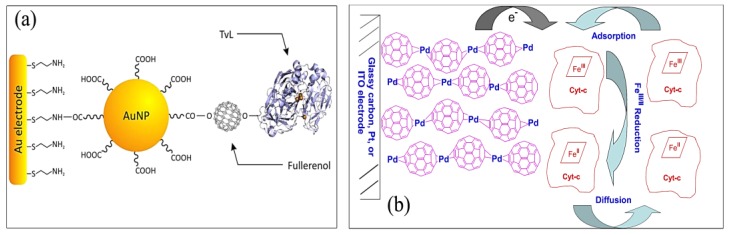
(**a**) Au-SAM/AuNPs-Linker/Fullerenols/TvL composite material assembly, adapted from [88] and (**b**) proposed mechanism of cyt *c* immobilization and electrochemical reduction by C_60_-Pd polymer film modified electrode, adapted from [89].

Lin *et al.* developed a mixed-valence cluster of cobalt(II) hexacyanoferrate and fullerene C_60_-enzyme-based electrochemical glucose sensor [82]. The C_60_-GOD was synthesized and applied with mixed-valence cobalt (II) hexacyanoferrate for analysis of glucose. Glucose in solution can be oxidized by C_60_-GOD-modified glassy carbon electrode, which is followed by the oxidation of the reduced C_60_-GOD by oxygen in the solution and formation of H_2_O_2_. On the other hand, the cobalt(II) hexacyanoferrate (Co_3_[Fe(CN)_6_]_2_)_(Red)_ can oxidize by means of produced H_2_O_2_. At the end, the oxidized Co_3_[Fe(CN)_6_]_2_)_(Ox)_ was reduced with an applied electrode voltage at 0.0 mV (*vs.* Ag/AgCl) and the reduced current can be traced for the detection of glucose (Scheme 4a).

It has been shown that the electrodes with immobilized enzymes (C_60_-GOD) shows better responses than the electrode with free enzyme. In addition, it was demonstrated that the electrode with a thicker cobalt (II) hexacyanoferrate coating produces a larger current response for the H_2_O_2_. However, the current response apparently tends to level off with larger amounts of cobalt (II) hexacyanoferrate coating. The C_60_- GOD/cobalt(II) hexacyanoferrate-modified electrode in solutions at a higher stirring rate exhibited a larger current response to the same concentration of glucose. However, the current response apparently tends to level off at a higher stirring rate. Moreover, an optimum current response is obtained around pH = 6.2 and 30 °C.

*Urease:* Urea is one of the byproducts of protein metabolism. The precise detection of urea is crucial in various biomedical applications, glomerular filtration rate determination, and renal function tests. The enzyme urease could be employed for urea determination, whereby the urease catalyzes the hydrolysis of urea to form alkaline reaction products of NH_4_^+^ and CO_3_^2−^. The detection is based on pH changes resulted from by enzymatic reaction [90]:

$${\text{NH}}_{2}{\text{CONH}}_{2}\;+\;H_{2}O\;\overset{Urease}{\to }\;{{\text{NH}}_{4}}^{+}\;+\;{{\text{CO}}_{3}}^{2-}$$

Fullerenes have been used in the fabrication of certain biosensors with enzymes such as lipase and urease. Integrating of fullerene molecule for construction of biosensing devices may enhance the sensitivity of the analytical method when it is combined with urease because it provides the high surface area-to-volume ratio for urease immobilization.

A new way to construct a urea potentiometric biosensor has been developed by Saeedfar *et al.* [91]. The fullerene nanomaterial was functionalized with carboxyl groups by sonication, heat, and ultraviolet (UV) radiation. *N*,*N*′-dicyclohexylcarbodiimide (DCC) or *N*-(3-dimethylaminopropyl)-*N*′-ethylcarbodiimide hydrochloride (EDC) was utilized to immobilized urease enzyme onto carboxylic modified fullerenes (C_60_-COOH). It was observed that the lower sensitivity of the urea biosensor was obtained using water-insoluble DCC as a cross-linking agent instead of EDC. The immobilized urease catalyzed the hydrolysis of urea in the sample, which resulted in the production of OH^−^ ions. When the concentration of urea is low, the OH^−^ ion reacted with buffer and the concentration of the buffer became important. Therefore, the buffer capacity could not maintain the pH and the sensitivity increased. When the concentration of buffer is high, the sensitivity of the biosensor decreased because of the OH^−^ ion reacted with buffer. The optimum pH range of the biosensor was obtained between pH 6.0 and 8.0. In another study, a Fullerene-C_60_-coated piezoelectric quartz crystal urea sensor based on either solvated or immobilized urease was developed and applied to detect urea in aqueous solutions. However, the immobilized C_60_-urease urea detection system shows lower sensitivity than that of the solvated urease detection (Table 1) [71].

*Laccase:* The high stability and bioactivity of the bio-electrochemical interfaces play a crucial role in the performance of laccase-based biosensors. The immobilization of enzymes on solid supports is one of the effective approaches meeting the requirement for a highly sensitive and stable biosensor. There is extensive interest to construct laccase biosensors in combination with nanomaterials, due to their unique properties [82]. The fullerene-C60 nanoparticles provide a suitable micro-environment for enzyme immobilization, maintaining their bioactivity, and accelerating the electron transfer between their redox active center and transducer surfaces [88]. An electrochemical biosensing platform based on the coupling of two different nanostructured materials (gold nanoparticles and fullerenols), has been developed and characterized by Lanzellotto *et al.* [88]. The proposed methodology was based on a multilayer material consisting in AuNPs, fullereneols, and *Trametes versicolor* laccase (TvL) assembled layer by layer onto a gold electrode surface (Scheme 4b).

A linear dependence has been obtained between the voltammetric peak currents and the potential scan rate which attributed to the immobilization of the redox protein. The calculated electron transfer rate constant (k_s_) values shows the higher amount of immobilized TvL on nanostructures-modified electrodes compared to the gold electrode due to the increased roughness of the electrode surface. It was believed that the presence of nanostructured material increases the protein loading due to high surface-active and provide an ideal microenvironment for proteins. Microscopic characterization of the electrode surface before and after modification with TvL has been performed by scanning tunneling microscopy. Before enzyme immobilization, several nanoparticles of 15 nm are observed and, after modification with TvL, a huge increase of particles size is detected (35 nm). In addition, it was observed that Michaelis constant (K_M_^app^) decreases after several introducing of AuNPs and fullerenol showing an increase of the enzymatic affinity for the substrate. It is ascribed to the fact that the fullerenes provide a suitable microenvironment for the protein immobilization and induce the protein molecule mobility in order to correctly orient its redox centers in order to achieve a proper electron transfer.

Single-walled carbon nanotubes (SWCN) and multi-walled carbon nanotubes (MWCN), with high surface area, high adsorption capacity, and rapid desorbability are widely used for construction of enzyme electrodes. One of the most recent example is reported by Barberis *et al.*, where simultaneous amperometric detection of ascorbic acid (AA) and antioxidant capacity has been performed based on fullerenes-C_60_/C_70_ or nanotubes-modified graphite sensor-biosensor systems, and ascorbate oxidase. It was found out that the combination of fullerene and ascorbate oxidase resulted in the complete AA shielding and in the highest selecting capacity toward AA while nanotubes only increase sensitivity without ability to discriminate between different compounds [92]. Authors hypothesized that fullerenes absorb more enzyme during dips, so that they can oxidize more AA before it reaches the transducer surface. 

#### 2.3.2. Redox Active Proteins

Direct electrochemistry of Hb immobilized on fullerene-nitrogen doped carbon nanotubes (C_60_–NCNT)/Chitosan (CHIT) composite matrix is reported by Sheng *et al.* [93]. The developed C_60_–NCNT/CHIT modified electrode was utilized for the determination of H_2_O_2_. TEM image of NCNT shows that after immobilization, some C_60_ amorphous nanoparticles with the size of ca. 4 nm were found visible inside NCNT. The obtained FTIR spectra showed that the relative shifts of the peaks which are ascribable to the π electron interaction between C_60_ and NCNT. The amide I and II bands related to Hb which is immobilized on C_60_–NCNT have similar shapes to that of free Hb indicating that Hb is successfully immobilized on C_60_–NCNT. No redox peaks were observed in the cyclic voltammograms of the bare GCE and the back-ground current increase at C_60_–NCNT/GC electrode. At Hb/NCNT/CHIT/GC electrode, there is only one cathodic peak which can be observed from the CV. After immobilization of Hb on the C_60_–NCNT/CHIT/GC electrode, a pair of well-defined redox peaks related to the Hb and (Fe^III^/Fe^II^) are observed.

*Cytochrome c:* Cytochrome *c* (cyt *c*) is a heme containing metalloprotein located in the inter membrane space of mitochondria. It has a low molecular weight (M_w_ = 12,400 D) with a single polypeptide chain of 104 amino acid residues covalently attached to the heme moiety. It plays a key role in biological respiratory chain, whose function is to transfer electrons between cytochrome *c* reductase (complex III) and cytochrome *c* oxidase (complex IV) [94].

One example of the application of the fullerene film modified electrodes for immobilizing a cyt *c* has been reported by D’Souza *et al.* [89]. Two types of fullerene film modified electrodes were utilized for immobilization of cyt *c*. One involves an electrochemically-conditioned fullerene drop-coated film electrode and the other an electro-polymerized fullerene, cross-linked with palladium acetate complex film electrode. The immobilization of cyt *c* on the fullerene film modified electrode was examined by piezoelectric microgravimetry at a quartz crystal microbalance (QCM). It was shown that upon addition of cyt *c* the frequency decreased to reach plateaus. In addition, the blue shift and the broadness of the bands observed in UV–Vis spectra was attributed to the cyt *c* molecules which are tightly packed on the electrode surface. The proposed mechanism of the cyt *c* immobilization is illustrated in Scheme 4c. It is believed that site *c* is immobilized by one or more of following ways: (1) electrostatically binding of the electron deficient C_60_ molecules with the cyt *c* molecules; (2) negative charge on the C_60_ film electrostatically bind with positively-charged parts of cyt *c* protein; and (3) surface structure of C_60_ or C_60_-palladium may affect the immobilization of cyt *c*.

The CV behavior of cyt *c* immobilized on the C_60_ drop-coated film GCE and the C_60_-Pd polymer film modified electrode shows that upon addition of cyt c to the solution, a cathodic peak appeared at Ep_c_ = −400 mV *vs.* Ag/AgCl. When the potential scan reverse, an anodic peak at Ep_a_ = 50 mV *vs.* Ag/AgCl was also observed indicating the reversible and slow electron transfer process. When the equilibrium occurs at C_60_-Pd polymer film-modified GCE in the cyt *c* buffer solution, the CV peaks were still present showing the stable immobilization of cyt *c* onto the C_60_-Pd polymer film-modified electrode. The effect of the C_60_-Pd polymer film thickness on CV properties of the immobilized cyt *c* was also examined and it is concluded from the obtained results that the amount of immobilized cyt *c* increased with the increase of the C_60_-Pd film thickness.

Csisza’r *et al.* utilized C_60_ fullerene film modified electrodes for the electrochemical reactions of cyt *c* [95]. They have investigated the electrochemical behavior of fullerene films in the neutral state, which are porous intrinsic semiconductors. They can be reduced to form semiconductor or conducting salts. They assumed that partially reduced fullerene films have a structure with a pole, or negatively-charged outside and an apolar inside. The porosity of the films was estimated in two ways: firstly, by means of measuring the oxidation of gold and the reduction of the oxide in phosphate buffer. The oxidation and reduction waves can be suppressed by the presence of fullerene films. The other method is based on chronocoulometry method which allows the calculation of the electrode surface area. It was assumed that partial reduction produces irreversibly small amounts of C_60_^−^, and/or C_60_^2−^ intermediates and the film becomes a cation exchanger. When partially-reduced fullerene films used, the electrochemical response of cyt *c* became much better. In the case of thin film, the half-wave potential of quasi-reversible reaction was 285 mV, which is close to the standard redox potential of native cyt *c* (260 mV). It was also shown that with thicker films, the catalytic activity of cyt *c* is lower. In addition, the presence of partially reduced fullerene films stabilized the electrochemical reaction of cyt *c*. The response on reduced and then oxidized films was also investigated. The oxidized film was still apparently coherent and did not show any signal of cyt *c*. A better response was observed if the films were porous and partially charged. Neutral fullerene films lack the charge, and fully reduced or oxidized films lack the porous character. If the fullerene film reduction was carried out in the presence of Na^+^, resulted in completely inactived electrode because it converts the films mainly to semiconducting Na_6_C_60_, which cannot participate in the reaction of cyt *c*. If the film reduction was carried out in the presence of K^+^, the electrodes showed short-lived transient responses again, as on bare electrodes or with neutral fullerene films.

**Table 1:** Comparison of different fullerene-C_60_ modified biosensors.

Receptor	Analyte	Linear Range	Sensitivity	LOD	References
ssDNA	Dopamine	2–160 μM	-	0.6 μM	[58]
ssDNA	PDGF-BB	0.002–40 nM	-	0.6 pM	[64]
ssDNA	Thrombin	1 μM–10 nM	-	0.3 fM	[65]
ssDNA	16S rDNA	-	-	-	[54]
dsDNA	CD	0.1–25.0 nM	0.0235 μA·nM^−1^	0.03 nM	[66]
dsDNA	dopamine	10^−5^–10^−2^ M	100 nA.nM^-1^	1.2 μM	[60]
dsDNA	Epinephrine	10^−6^–10^−2^ M	100 nA.nM^-1^	0.1 μM	[60]
dsDNA	Norepinephrine	10^−5^–10^−2^ M	0.1 nA.nM^-1^	2.3 μM	[60]
Anti-IgG	IgG	-	1.25 × 10^2^ Hz/(mg/mL)	-	[69]
Anti-Hb	Hb	-	1.5 × 10^4^ Hz	<10^−4^ mg/mL	[69]
Anti-*E. coli*	*Escherichia coli* O157:H7	3.2 × 101 to 3.2 × 106 CFU/mL	-	15 CFU/mL	[72]
GOD-Chit	Glucose	0.05–1 mM	-	694 ± 8 μM	[80]
cobalt(II) hexacyanoferrate-GOD	Glucose	0–8 mM	5.60 × 10^2^ nA/mM	1.6 μm	[82]
Glucose oxidase	Glucose	-	5.9 × 10^2^ Hz/Δlog M	3.9 × 10^−5^ M	[87]
Urease	Urea	1.2 mM–0.042 mM	59.67 ± 0.91 mV/dcade	-	[91]
AuNPs-TVL	Laccase	0.03–0.30 M	-	0.006 mM	[88]

## 3. Conclusions and Future Prospective

Recently, nanostructured materials have been significantly used to create state-of-the-art electrochemical biosensors with enhanced performance. They provide the analytical devices with the ability of miniaturization and reduced response time, and cost effectiveness for application in clinical diagnosis. Among different nanomaterials, carbon nanomaterials hold potential promise as a material for designing a new generation of biosensors due to their unique characteristics. Recently, fullerene-C_60_ contributed greatly to the field of biosensing and bio-nanotechnology. The unique electrochemical and physicochemical properties, together with biocompatibility characteristics of fullerene, allow its wide use for designing the highly sensitive chemical/biosensors.

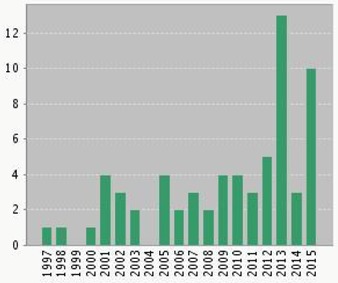
Published items in each year (www.webofknowledge.com).



In this review, we presented the most recent applications of fullerene-C_60_ based electrochemical biosensors which employed various kinds of biomolecules. Especially, electrochemical biosensors based on the interaction between fullerene-C_60_ and DNA has been reviewed in depth. It has been shown that fullerene-C_60_ has been widely utilized for improving the sensitivity of electrochemical biosensors. Not only they provide a suitable immobilization platform for DNA and antibodies, but they also have the ability to induce in redox-active proteins a proper orientation which leads to better electron transfer properties. Therefore, fullerene-C_60_ can be easily extended to immobilize and obtain direct electrochemistry of enzymes and proteins. 

However, the range of applications is still limited and further investigation is required. Easy functionalization and high surface area of fullerene can be utilized for designing more sensitive biosensing devices with high stability. The recent developments of electrochemical biosensors based on fullerene-C_60_ may bring many researchers to use other analogues of fullerene-C_60_ in the construction of electrochemical biosensors. Furthermore, multiple functionalizations of these kinds of nanomaterials may lead to the improved performance of biosensors. On the other hand, taking into account the biocompatibility of fullerene-C_60_, different kind of biomolecules such as microoganisms, organelle, and cells can be easily integrated in the biosensors fabrication. Moreover, fullerene-based biosensors could be integrated within bio-chips with on-board electronics. This will lead to fabricating devices which are small, low-cost, with simple operation procedure. Therefore, electrochemical biosensors based on fullerene-C_60_ with their cost-effectiveness and suitability for microfabrication can be expected to become increasingly popular in the near future.
